# Trajectories of Serum Albumin Predict Survival of Peritoneal Dialysis Patients: A 15-year Follow-Up Study

**DOI:** 10.1097/MD.0000000000003202

**Published:** 2016-03-25

**Authors:** Ping-Fang Chiu, Chun-Chieh Tsai, Chia-Lin Wu, Tse-Yen Yang, Hung-Hsiang Liou, Hung-Lin Chen, Chew-Teng Kor, Chia-Chu Chang, Horng-Rong Chang

**Affiliations:** From the Institute of Medicine (P-FC, H-RC); School of Medicine (P-FC, C-CC, H-RC), Chung Shan Medical University, Taichung; Division of Nephrology, Department of Internal Medicine (P-FC, C-CT, C-LW, H-HL, C-CC), Changhua Christian Hospital, Changhua; Molecular and Genomic Epidemiology Center (T-YY), China Medical University Hospital, Taichung; Division of Nephrology (H-HL), Department of Internal Medicine, Hsin-Jen Hospital, New Taipei City (H-LC); Department of Nutrition and Dietetics; Internal Medicine Research Center (C-TK), Changhua Christian Hospital, Changhua; PhD. Program for Aging (C-CC), College of Medicine, China Medical University; and Division of Nephrology (H-RC), Department of Internal Medicine, Chung Shan Medical University Hospital, Taichung, Taiwan.

## Abstract

Although initial serum albumin level is highly associated with overall and cardiovascular mortality in peritoneal dialysis (PD) patients, we consider that the dynamic change and trend of albumin after initiation of PD are also essential.

We enrolled patients who received PD for more than 3 months from January 1999 to March 2014. We categorized these patients into 2 groups by the difference in serum albumin level (Δalbumin = difference between peak with initial albumin level = peak albumin level − initial albumin level) after PD. The patients with Δalbumin < 0.2 g/dL (median level) were considered as group A (n, number = 238) and those with Δalbumin ≥ 0.2 g/dL were considered as group B (n = 278). Further, we stratified these patients into quartiles: Q1 Δalbumin < −0.2 g/dL; Q2, −0.2 ≦∼ <0.2 g/dL; Q3, 0.2 ≦∼ <0.6 g/dL; and Q4, ≥0.6 g/dL. Regression analysis was performed to determine the correlation of initial albumin and Δalbumin.

Group A patients presented with higher levels of serum albumin (3.71 ± 0.54 vs 3.04 ± 0.55 g/dL; *P* < 0.001) and hematocrit as well as better initial residual renal function. However, those in group A had lower serum albumin increment and downward-sloped trends after dialysis. In contrast, the albumin trend was upward sloped and the increment of albumin was remarkable in group B, despite the high prevalence of cardiovascular diseases and diabetes. Overtime, group A patients had poorer survival and experienced more frequent and longer hospitalizations. Group Q1 patients with least albumin increment had worst survival. Group Q4 patients with lowest initial albumin also had poor survival. Age, diabetes, cardiovascular diseases, BMI, initial albumin, and Δalbumin could affect patient outcomes independently. Regression analysis showed a better outcome can be obtained if the initial albumin level is at least above 3.15 g/dL. (Initial albumin level = −0.61 × Δalbumin + 3.50.)

The increment and trend of albumin especially during early period of PD may be a more crucial determinant for survival.

## INTRODUCTION

Serum albumin level and nutritional status are important survival determinants and indicators for patients receiving hemodialysis or peritoneal dialysis (PD).^1^ In patients receiving PD, the initial serum albumin levels are closely related to cardiovascular mortality,^2,3^ PD technique survival,^4^ and peritonitis rate.^5,6^ Serum albumin levels also reflect conditions, including inflammation,^7^ dialysis adequacy, residual renal function,^8,9^ and volume status in PD patients.^10^

Lower protein diet is widely applied to slow renal deterioration in patients with chronic kidney disease (CKD), and malnutrition may not occur in patients who follow a well-planned diet as observed in a study.^11^ However, patients with CKD stage 5 are frequently malnourished, which is caused because of dietary protein over-restrictions by patients’ themselves.^12^ Ideally, dialysis modality corrects uremia and metabolic acidosis and improves nutrition. The serum albumin level will rise to a steady state gradually^13^ and achieve the peak level. It has been demonstrated that the changes of albumin level in the fixed postdialysis period were associated with the survival of patients receiving PD.^14^ After the inflection point of peak albumin and with ongoing dialysis, the albumin level will decline progressively because of various reasons, for example, worsening renal reserve and decreasing dialysis adequacy and increasing uremia and dialysis complications including infectious and cardiovascular diseases.^15^

Although the initial serum albumin level is important to predict outcomes in patients receiving PD, we assume that the dynamic change and trend in albumin level may reflect the status of PD more accurately. The correlation between the initial and dynamic changes of serum albumin should also be comprehended. Furthermore, we also try to find the critical level of albumin for better outcome obtained after initiation of PD.

## METHODS

We enrolled all patients receiving PD for more than 3 months between January 1999 and March 2014 in Changhua Christian Hospital. Those who had incurable malignancy before dialysis, kidney transplantation, advanced liver cirrhosis (Child–Pugh score ≥ B), and died from accidents were excluded. Basic characteristics of the patients including serum albumin levels and other biochemical test, associated comorbidities, peritoneal equilibration test, dialysis adequacy, and residual renal clearance were collected and analyzed. The impactions of serum albumin on the patients’ survival were investigated separately following PD initiation, during PD steady state (peak albumin level), and end of PD. In addition, we categorized these patients into 2 groups based on the median level of the increment in serum albumin (Δalbumin = difference between peak with initial albumin level = peak albumin level − initial albumin level) following initiation of PD. In this study, we analyzed the effect of various cut-points on mortality risk using same adjusted Cox PH regression model as Table 2. Results revealed that significance reduces mortality risk (adjusted hazard ratios [HRs] were 0.53–0.64) if Δalbumin cut-point was setup at between −0.4 and +0.2, and these adjusted HR were consistent (heterogeneity test: I2 = 0%, *P* = 0.992). However, if the cut-point was setup at below −0.5 and above 0.3, there were no significance effects on mortality. Therefore, value +0.2 (the median value of Δalbumin at the same time) was chosen as the cut-point for grouped patient. Group A included patients with Δalbumin <0.2 g/dL, and group B included patients with Δalbumin ≥0.2 g/dL.

**TABLE 2:**
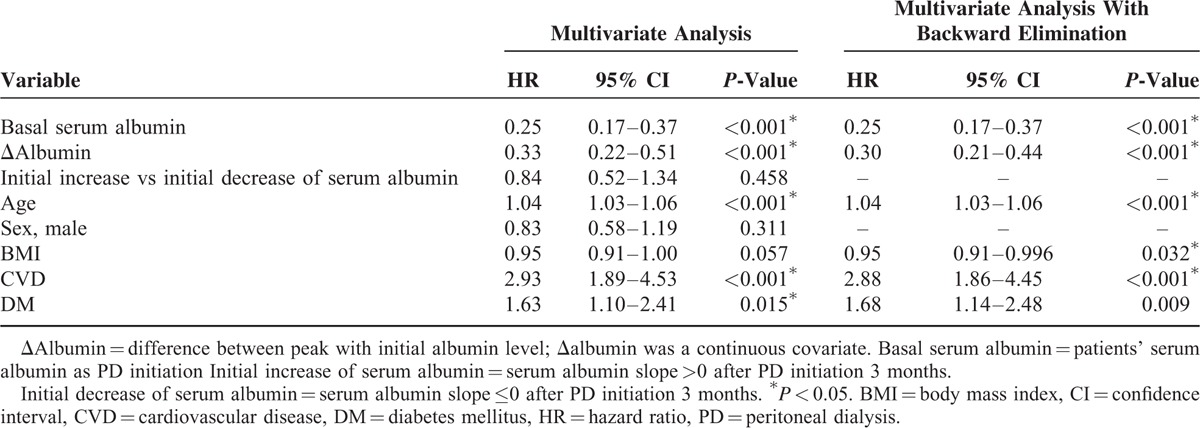
Cox Proportional Hazard Regression: Hazard Ratio of Variables and Survival

**FIGURE 1:**
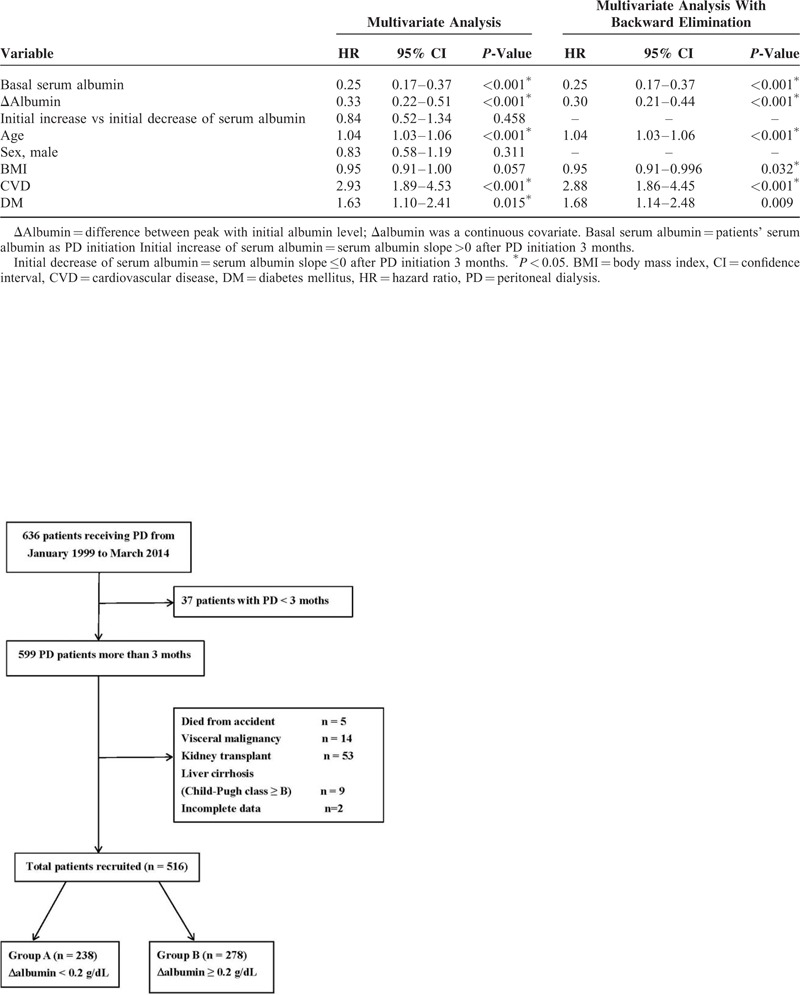
Participant flow diagram depicting the screening/enrollment process.

Furthermore, in order to clarify the correlation of initial levels and increment of albumin, these patients were stratified into quartiles according to Δalbumin: quartile 1 (Q1) Δalbumin < −0.2 g/dL; quartile 2 (Q2), −0.2 ≦∼ <0.2 g/dL; quartile 3 (Q3), 0.2 ≦∼ <0.6 g/dL; and quartile 4 (Q4), ≥0.6 g/dL. The HRs were computed and compared with Q1 group. The distribution of primary renal diseases, comorbidities, education level, occupation, and baseline characteristics were also comparable between these 4 groups. Major events requiring hospitalization occurring after initiation of PD were also surveyed. Bromocresol purple dye method was adopted for laboratory albumin measurement. To improve the quality of reporting in observational studies, the manuscript was organized in a manner compliant with the Strengthening the Reporting of Observational Studies in Epidemiology statement.^16^ The patient flow chart is shown in Figure 1, which includes the number of patients recruited and excluded from the study. The approval of the Institutional Review Board of the Changhua Christian Hospital was obtained (IRB No.140516).

### Statistical Analyses

The continuous and categorical variable differences between the groups were analyzed using the Student's *t*-test, Mann–Whitney *U*-test or Chi-squared test. A linear mixed model was used to analyze the continuous change in serum albumin level. The *P* values for trend were calculated by the Jonckheere–Terpstra test to assess linear trends across varying time. Kaplan–Meier estimation and log-rank test were performed for survival analysis. Cox proportion hazard regression analysis was also conducted to assess the possible confounding factors. HRs and 95% confidence interval were calculated in these models. Linear regression analysis was carried out for determining the correlation of initial albumin and albumin difference. All statistical analyses were performed by using the SAS statistical package (version 9.4 for Windows; SAS Institute, Inc., Cary, NC).

## RESULTS

**FIGURE 2:**
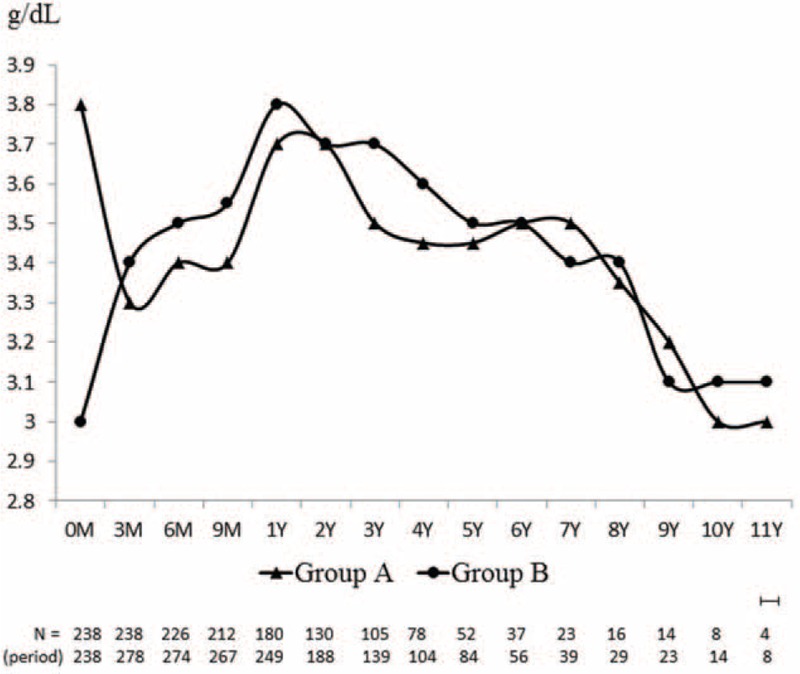
Trends of median values of albumin illustrated after initiation of peritoneal dialysis (PD). Dash line indicates the turning point of trajectory. At year 1, the peak level of serum albumin achieved after PD. Baseline value between group A and B were significantly different (3.8 vs 3.0 g/dL; *P* < 0.001^a^). However, the difference of value disappeared at 1 year (3.7 vs 3.8 g/dL; *P* = 0.09^a^). From 0 to 1 year, group B had an upward slope, but not for group A. Comparing to group A, the slope of group B was significantly greater (−0.1 vs +0.6 g/dL/year; *P* < 0.001^b^). The *P* for trends was 0.067 and <0.001^c^ in group A and B, respectively. After 1 year, both groups had decreased trends with significant *P* for trends (0.012^c^ vs 0.001^c^). Their slopes were not significantly distinct as well (−0.07 vs −0.09 g/dL/year; *P* = 0.37^b^). a, calculated by Mann–Whitney *U*-test; b, calculated by linear mixed model; and c, calculated by Jonckheere–Terpstra test.

A total of 516 patients (278 females, 238 males) were included in the study. The baseline characteristics of all the patients are shown in Table 1. The initial albumin level of all the patients was 3.35 ± 0.64 g/dL, the peak albumin level was 3.7 ± 0.34 g/dL, and the end-PD albumin level was 2.92 ± 0.74 g/dL. The mean albumin levels at different time points, including initial, peak, and end of PD, were related to final survival (HR = 0.62, 0.34, and 0.57, respectively). Group A included 238 patients with Δalbumin < 0.2 g/dL, and group B included 278 patients with Δalbumin ≥ 0.2 g/dL. The time-lapsed median levels of albumin for groups A and B are separately shown in Figure 2. Baseline value between group A and B were significantly different. However, this statistic difference disappeared at year 1, the peak, and turning point of albumin trajectory. From 0 to 1 year, group B had an upward slope, but not for group A. The slope of group B was significantly greater than that of group A. The *P* for trend was also significant in group B. After 1 year, both groups had decreased trends with significant *P* for trends. But their slopes were not significantly distinct.

**TABLE 1:**
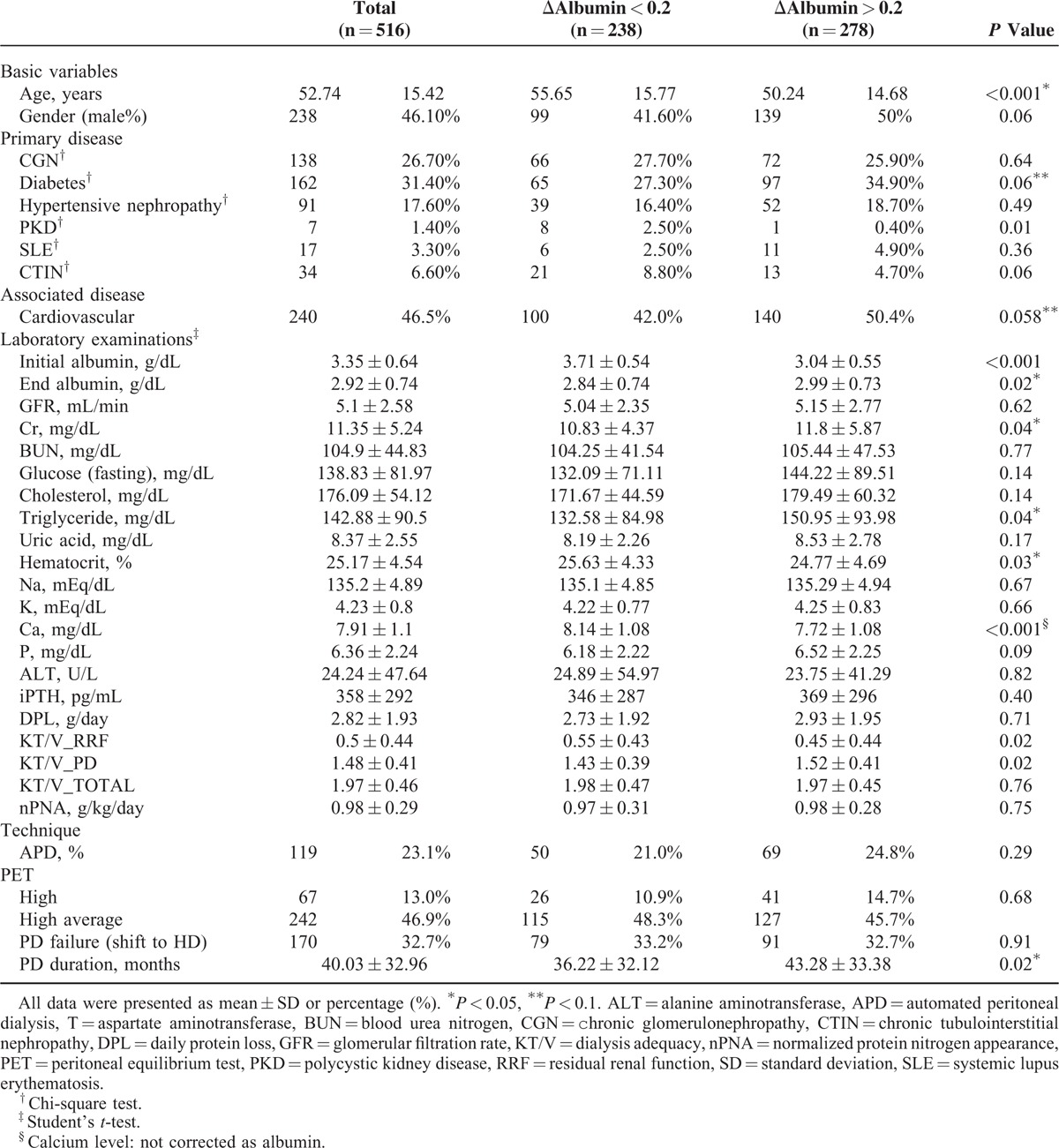
Overall Characteristics of Peritoneal Dialysis Patients

The initial mean albumin levels were 3.71 ± 0.54 g/dL in group A patients and 3.04 ± 0.55 g/dL in group B patients (*P* < 0.001, Table 1). Nevertheless, group A had less albumin increment after initiation of PD. At the end of PD, the group A patients had lower serum albumin levels due to PD termination or mortality (Table 1). In contrast, group B patients had more increment of albumin. After adjustment using multivariate analysis, group B patients had superior survival. A Cox regression model was used to estimate the HR, so as to identify the factors, namely older age, preexisting diabetes, cardiovascular diseases, body mass index (BMI), initial albumin, and Δalbumin, could affect patient outcomes independently (Table 2). However, initial increase or worsening of albumin after PD initiation 3 months could not affect the patients’ survival.

The rate and duration of hospitalization from initiation to steady PD state were analyzed and are presented in Table 3. Overall, the hospitalization rate was highest during the 1st year (61%) after PD initiation. The rate diminished later but increased again in the 5th year after initiation of PD (29.4%, 20.2%, 14.6%, 12.1%, 16.6%; 2nd–5th year, respectively). Group A patients underwent more and longer hospitalizations in early PD period (Table 3). Hospitalizations were resulted due to various causes, including cardiovascular disease, cerebrovascular disease, infection, and mechanical problems (catheter migration or obstruction, abdominal hernia, dialysate leakage, etc.). There was no significant difference of the attributed cause of hospitalization between the 2 groups. When compared with group A, group B patients also stayed longer in PD (Table 1).

**TABLE 3:**
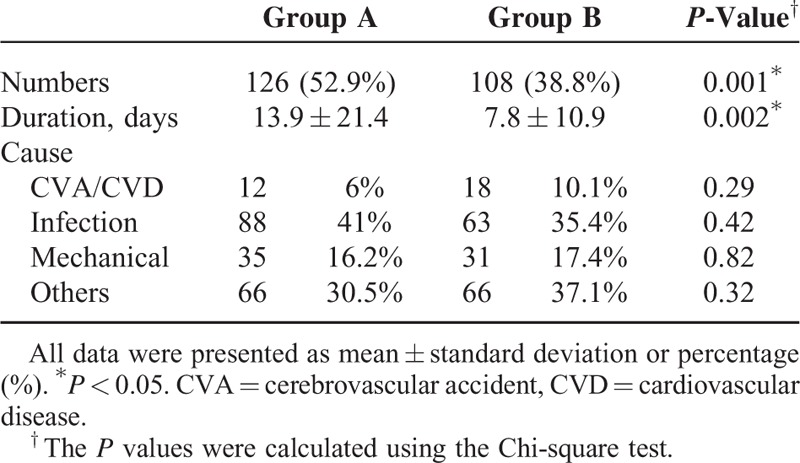
Duration and Causes of Hospitalization During the Designated Period

**FIGURE 3:**
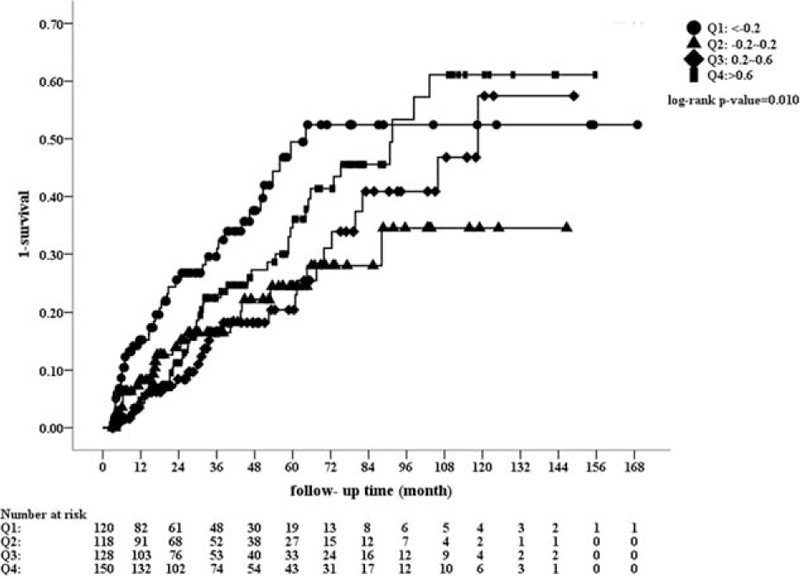
Risk curve of quartile as albumin difference (Δalbumin). Groups Q1 patients with least albumin increment and Q4 patients with lowest initial albumin had poor survival after PD initiation when compared with groups Q2 and Q3.

**FIGURE 4:**
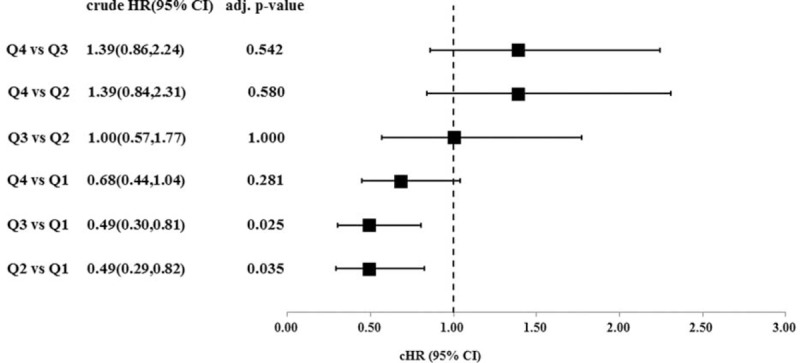
Pairwise comparison of risk in quartile groups. When compared with Q1 patients, the hazard ratio was reduced in groups Q2 and Q3 separately. For Q4 patients, the benefit from albumin gain was no longer obtained. (The pairwise comparison across each quartile of Δalbumin was performed by using Tukey's method and the corresponding adjusted *P*-value was calculated.).

**FIGURE 5:**
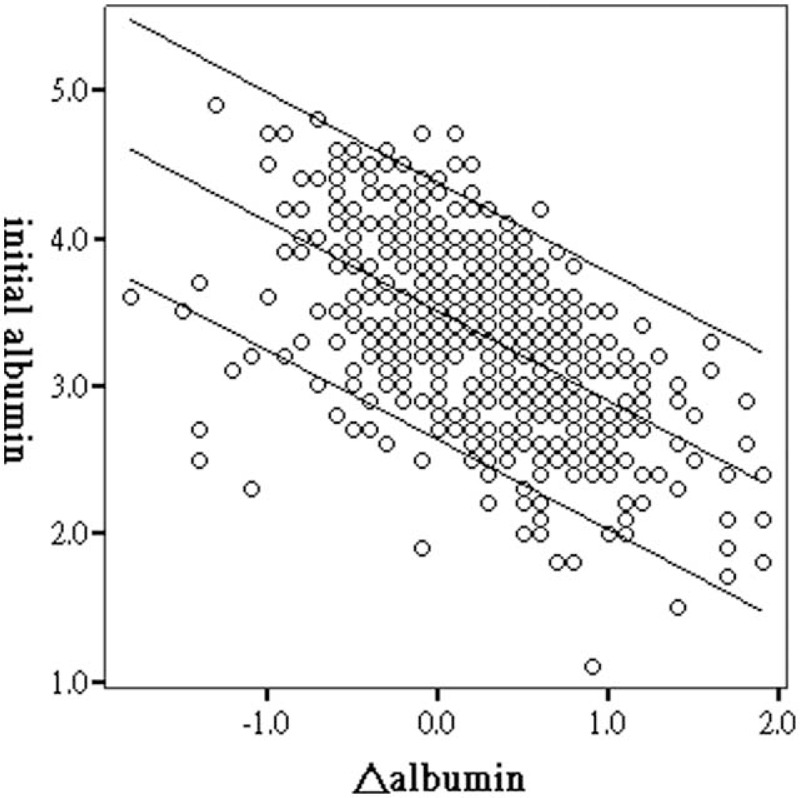
Scatter plot and linear regression. Correlation with initial albumin versus Δalbumin. Initial albumin level = −0.61 × Δalbumin + 3.50 (*R* = 0.57; *P* < 0.001).

After further stratification of these patients into quartiles based on Δalbumin, the baseline characteristics were comparable between these 4 groups with exception of age and initial albumin level. Group Q1 patients were the oldest and Q4 patients were the youngest (Q1: 59.2 ± 14.6, Q2: 56.0 ± 16.1, Q3: 51.6 ± 14.5, and Q4: 49.1 ± 14.7 year-old; *P* < 0.001). The initial serum album levels of 4 groups are, respectively, described as follows: group Q1: 3.77 ± 0.59 g/dL, Q2: 3.63 ± 0.49 g/dL, Q3: 3.30 ± 0.52 g/dL, and Q4: 2.82 ± 0.49 g/dL, *P* < 0.001. The risk curve of plot for mortality was drawn as shown in Figure 3. When compared with Q1 patients, the HR was reduced in groups Q2 and Q3 (Figure 4). For Q4 patients, the benefit for survival from albumin gain was no longer maintained. The correlation of initial albumin and Δalbumin was computed and is demonstrated in Figure 5. The formula was: initial albumin level = −0.61 × Δalbumin + 3.50. For a uremic patient considering PD as renal replacement therapy to obtain the better survival benefit from albumin gain, the lowest initial albumin level is not less than 3.15 g/dL.

## DISCUSSION

Serum albumin level represents the sum of protein and calorie intake, dialysis adequacy, peritoneal and renal albumin loss, concurrent illness, and underlying systemic disease. Previous longitudinal studies also showed that lower initial serum albumin levels are associated with cardiovascular disease in the general population.^17,18^ Albumin levels at the beginning of dialysis also affect the PD patients’ survival.^2^ In our study, albumin levels at different time points, including at the beginning, steady state, and at the end of PD, are associated to overall survival as well. The finding is consistent with our belief, but nevertheless, the peak level of albumin seemed to be more relevant for patients’ outcome. It implied that the trend or increment of albumin is worthwhile to be investigated.

In our study, groups A and B patients have similar albumin trajectory after achieving peak level of serum albumin. However, there is quite different in the initial albumin trends and slopes between the 2 groups. Group A patients had higher initial albumin level, hematocrit, better residual renal function, but got lower increment in albumin levels after PD. In contrast, the group B patients had initial lower albumin levels but gained steady increase following PD initiation and achieved higher increments and peak of albumin finally. Overtime, group A patients had poorer survival. We also found a high risk for hospitalization during the early PD period. They experienced deteriorative course and encountered more frequent hospitalizations during this designated period, especially elder patients. Group B patients had superior survival eventually, even though they had higher prevalence of cardiovascular disease and diabetes. The result is also valid after adjustment of several confounding factors which influencing patients’ survival including age, cardiovascular disease, and diabetes. Moreover, the higher prevalence of cardiovascular and diabetes may account for end-stage renal disease in group B patients at a younger age.

Protein–energy malnutrition always develops during the course of CKD. Patients with advanced CKD spontaneously reduce their mean protein and energy intake.^19^ For patients with CKD stages 3–5, strict protein control is advised to slow renal disease progression, decrease serum potassium and phosphate, and reverse metabolic acidosis.^20^ Upon dialysis initiation and stabilization, the general condition and nutritional status constantly improve.^21^ Almost several months after dialysis, the steady state and peak serum albumin level are achieved. However, some patients had experienced adverse events due to various illnesses during their early dialysis period. These unfortunate situations counterbalance the benefits from dialysis. Mechanical problems such as catheter migration and dialysate leakage, dialysis-related or -unrelated infections, and psychosocial and emotional instability may occur during the early PD period. Hence, both improvements and deteriorations may occur among patients receiving PD. Nevertheless, the advantage from increments of albumin will be limited if there is excessive malnutrition at the beginning. For a PD patient with achievement of maximal albumin gain (0.6 g/dL) after PD, if better survival will be obtained, the lowest initial albumin level is 3.15 g/dL.

The impact of age on patients’ survival should be further clarified. In clinical practice, it is often that younger PD patients may have more major comorbidities, which lead to uremic state in their youth. However, they have more potential to gain benefit from dialysis and avoid adverse events resulting from superior self-caring ability. However, very low albumin status that represents poor general condition restricts the improvement even though the patients might be of a younger age. Age, comorbidity, and initial levels and increment of albumin all contribute to patients’ survival, despite their close correlation. The impact of increment of albumin still exactly existed after adjustment in the multivariate analysis. BMI is also a confounding factor on mortality in dialysis patients. Interestingly, patients with higher BMI had lower mortality trend in our study. It is regarding as “reverse epidemiology” and consistent with most studies in hemodialysis population_._^22^

In addition to a nutritional marker of serum albumin level, there are many factors that contribute to hypoalbuminemia: inflammation/infection and oxidative stress,^23,24^ uremia toxicity, hyperparathyroidism,^25^ fluid overload, renal or peritoneal albumin loss, metabolic acidosis, psychosocial factors, dysregulation of anorexigenic hormones leptin and ghrelin,^26^ as well as resistance of insulin.^27^ In patients undergoing PD, protein intake greater than 0.94 g/kg/day favors improved nutritional status and long-term outcomes.^28^ Our patients in the study almost met the minimal daily protein requirement after dialysis.

The aim of this study was not to diminish the predictive valve and the impact of albumin level, hematocrit, and residual renal function on PD patients’ survival. On the contrary, well-planned diet control in CKD stage 5 (for better initial albumin) and continuously attentive care following PD (for better Δalbumin) should be stressed. According to Figures 3 and 4, the least increment of albumin in Q1 group had highest mortality after PD initiation. Consequently, the medical team should make efforts to facilitate the patients’ health and avoid complications and hospitalization during this vulnerable period.

Our study has some limitations. The study sample size was relatively small, the analysis was single center retrospective, the demographic studied was narrow, and the results cannot be generalized. However, from this long cohort observation study, it is worth mentioning that combined initial albumin level and its gradient during the designated period are predictive for outcomes. Possible biases including survival-related bias, rates of mortality and hospitalization in individual hospital, various causes of predialytic hypoalbuminemia, and modality of dialysis may affect the outcomes. To limit the influences, we excluded the patients with incurable cancer because the advancing anticancer therapy in recent years and patients with severe cirrhotic liver from its profound and rigid impact on serum albumin. Moreover, risks of mortality and hospitalization in our patients were close to others inquired from United States Renal Data System.

In conclusion, serum albumin is critical and predictive for patients’ survival, regardless of the dialysis state. Additionally, the albumin increment and its trend after dialysis are also important in this aspect. Therefore, by maintaining well-planned diet control in CKD stage 5 and continuously comprehensive care following PD, the patients can ultimately have better survival.
